# Xenograft and organoid model systems in cancer research

**DOI:** 10.15252/embj.2019101654

**Published:** 2019-07-08

**Authors:** Margit Bleijs, Marc van de Wetering, Hans Clevers, Jarno Drost

**Affiliations:** ^1^ Oncode Institute Princess Máxima Center for Pediatric Oncology Utrecht The Netherlands; ^2^ Oncode Institute Hubrecht Institute Royal Netherlands Academy of Arts and Sciences and University Medical Center Utrecht The Netherlands

**Keywords:** cancer, organoids, preclinical models, tumour heterogeneity, xenografts, Cancer, Molecular Biology of Disease, Synthetic Biology & Biotechnology

## Abstract

Patient‐derived tumour xenografts and tumour organoids have become important preclinical model systems for cancer research. Both models maintain key features from their parental tumours, such as genetic and phenotypic heterogeneity, which allows them to be used for a wide spectrum of applications. In contrast to patient‐derived xenografts, organoids can be established and expanded with high efficiency from primary patient material. On the other hand, xenografts retain tumour–stroma interactions, which are known to contribute to tumorigenesis. In this review, we discuss recent advances in patient‐derived tumour xenograft and tumour organoid model systems and compare their promises and challenges as preclinical models in cancer research.

## Introduction

**Figure 1:**
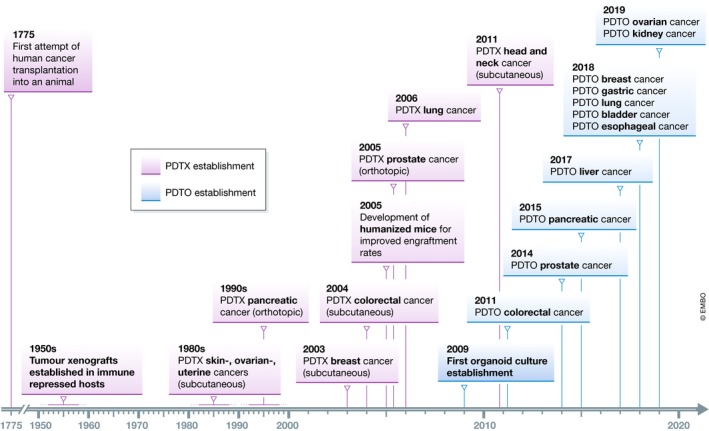
Timeline PDTX and PDTO development (Toolan, 1953; Taetle *et al*, 1987; Fu *et al*, 1992; Beckhove *et al*, 2003; Fichtner *et al*, 2004; Shultz *et al*, 2005; Wang *et al*, 2005; Cutz *et al*, 2006; Sato *et al*, 2009, 2011; Hennessey *et al*, 2011; Gao *et al*, 2014; Karthaus *et al*, 2014; Boj *et al*, 2015; Lee *et al*, 2018; Li *et al*, 2018, 2019; Sachs *et al*, 2018; Yan *et al*, 2018; Kopper *et al*, 2019; Schutgens *et al*, 2019).

Cancers consist of a continuously evolving heterogeneous cell mass (McGranahan & Swanton, 2017). Importantly, not all cells within a tumour contribute equally to its progression. Early studies on mouse mammary tumours revealed that cellular subpopulations from different regions of the same tumour vary in growth rate, drug response, immunogenicity and metastatic capacity (reviewed in Heppner, 1984; Tabassum & Polyak, 2015). This intra‐tumour heterogeneity can arise from both genetic and non‐genetic variability within tumours, such as variations in availability of resources, like differential access to oxygen and nutrients (Kreso & Dick, 2014; Tabassum & Polyak, 2015). The development of preclinical model systems phenocopying tumour heterogeneity is required for studying its contribution to tumour progression and acquisition of therapy resistance. Whereas the first patient‐derived tumour xenograft (PDTX) models were successfully established during the fifties (Toolan, 1953), patient‐derived tumour organoid (PDTO) models have been established only during the last decade (Sato *et al*, 2011) (Fig 1). Both PDTX and PDTO model systems are able to recapitulate the intra‐ and inter‐tumour heterogeneity seen in human cancers (Beckhove *et al*, 2003; Guenot *et al*, 2006; Huang *et al*, 2015; van de Wetering *et al*, 2015; Bruna *et al*, 2016; George *et al*, 2017; Nanki *et al*, 2018; Sachs *et al*, 2018; Yan *et al*, 2018). Therefore, these models are promising tools to study sub‐clonal dynamics within individual tumours during progression and therapy resistance (Shi *et al*, 2014).

**Figure 2:**
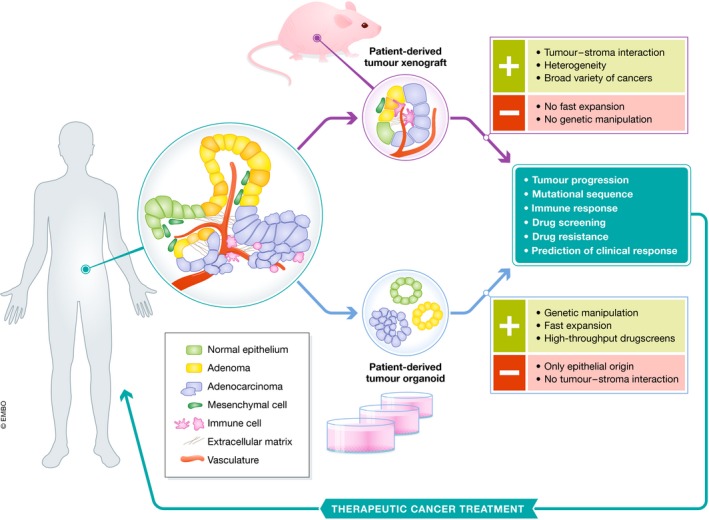
Schematic representation of patient‐derived tumour xenografts and organoids PDTXs preserve tumour heterogeneity and tumour–stroma interactions. PDTOs grow in a provided basement membrane extract and can be established from epithelial cancer cells as well as normal epithelial tissue. Both models allow for several translational applications that contribute to development of therapeutic cancer treatments. Part of this figure was adapted from Sachs and Clevers (2014).

Due to the complexity of human tumours, response to clinical cancer treatments varies substantially. Additionally, mechanisms of tumour progression are poorly defined as well as drug efficacy and resistance. While a high number of anti‐cancer compounds tested for clinical safety in Phase I studies progress to Phase II efficacy testing, most of these compounds fail in Phase II and III studies, which examine the power of pharmacological responses (Dimasi *et al*, 2013). Such high failure rates in clinical trials headline the need of preclinical efficacy models for improved predictions of clinical outcome. Several human preclinical models are currently used, including cancer cell lines, PDTX and PDTO cultures (Figs 1 and 2). These models have improved our understanding of the mechanisms of cancer progression and provided valuable tools for the development of novel cancer treatments. Additionally, these preclinical models are used to predict clinical response to anti‐cancer agents. In this review, we discuss PDTX and PDTO model systems and compare their promises and challenges in cancer research.

### PDTX models

To understand cancer biology and its translation into effective treatments, human preclinical models capturing the heterogeneity of cancer are fundamental. Although with low efficiency, primary tumour tissues can be grown in 2D cultures *in vitro*, allowing tumour cells capable of adapting to these conditions to expand and form a cell line. The use of *in vitro* cancer cell lines has provided valuable insights on tumour development and mechanisms of therapeutic actions (Sos *et al*, 2009; Greshock *et al*, 2010). However, the main drawback of cancer cell lines is the lack of both phenotypic and genetic heterogeneity found in the original tumours (Sachs & Clevers, 2014; Byrne *et al*, 2017). To enhance the correlation with human cancers, surgically derived primary clinical tumour samples can be grafted into mice, known as PDTX. In such models, tumour architecture and the relative proportion of cancer cells and stromal cells are maintained to a large extent, which yields better resemblance to the original tumour compared to cancer cell lines (Byrne *et al*, 2017; Fig 2). Although not all patient‐derived tumours can successfully be engrafted into mice, the success rate of PDTX establishment is increasing due to the establishment of immunocompromised mice (Shultz *et al*, 2005; Drake *et al*, 2012).

**Figure 3:**
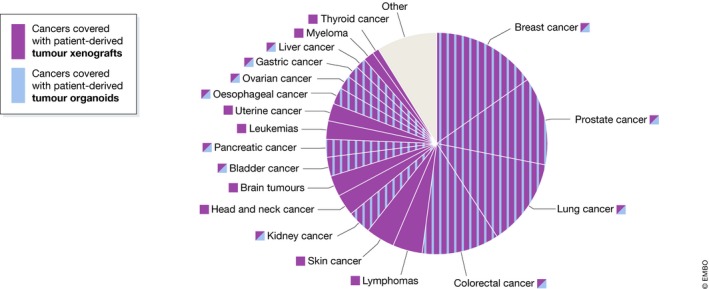
Pie chart with the different cancer types that can be grown as PDTX (left) and PDTO (right) marked in green In general, the engraftment efficiency of PDTXs is lower than the success rate of PDTO establishment.

Although the first documented attempt to transplant a human cancer into an animal dates back to 1775, a hallmark study by Helene Toolan showed that it was possible to grow human tumour cells in x‐irradiated mice and rats (Toolan, 1953). Additionally, she demonstrated that proliferation extended considerably when the x‐radiated hosts were treated with the immune system suppressor cortisone (Toolan, 1953). Later, Phillips and Gazet were able to obtain a slightly higher percentage of viable patient‐derived tumour grafts by treating the host mice with anti‐lymphocyte serum. The number of viable grafts increased in particular when combined with thymectomy, demonstrating that a suppressed immune response enhances engraftment efficiency (Phillips & Gazet, 1970). Following these studies, several genetically modified mouse models have been established, which are severely immune deficient, such as the NOD/SCID/IL2Rγ_null_ (NSG) mouse (Shultz *et al*, 2005). The virtual absence of an immune system in these mice allows for significantly higher engraftment rates (Shultz *et al*, 2005; Byrne *et al*, 2017). Together these studies demonstrated that loss of immune system activity improves engraftment and viability of patient‐derived tumour tissue into mice. PDTX models are currently established for a broad variety of cancers, including colorectal (Fichtner *et al*, 2004; Guenot *et al*, 2006), pancreatic (Fu *et al*, 1992; Kim *et al*, 2009), breast (Beckhove *et al*, 2003; Bruna *et al*, 2016), lung (Cutz *et al*, 2006), skin (Taetle *et al*, 1987), head and neck (Hennessey *et al*, 2011), prostate (Wang *et al*, 2005) and ovarian cancer (George *et al*, 2017; Fig 3). Although PDTXs recapitulate tumour tissue more closely than cancer cell lines, they are usually generated from a small amount of tumour material. As a consequence, the PDTX derived from it might not capture the full heterogeneity of the original tumour (Kemper *et al*, 2015). Moreover, Morgan and colleagues recently reported that from the total number of mutations detected in primary non‐small‐cell‐lung cancer (NSCLC) tumours, only 43% were detected in the corresponding PDTXs and four additional mutations arose in early passages of PDTXs that were not present in the primary tumour (Morgan *et al*, 2017). These observations suggest that clonal selection and evolution may occur early on upon tumour tissue engraftment into mice. Multiple biopsies from different regions of a tumour should be engrafted to capture the complete tumour architecture *in vivo*, whereas early passages of PDTXs should be used for translational applications to avoid outcomes that deviate from clinical response. Furthermore, the limited engraftment rates of PDTXs remain a major challenge, which is highly variable among cancers (Rosfjord *et al*, 2014). Sub‐clones of advanced tumours grow best as PDTX, compared to less advanced tumours. Additionally, growth rates of engrafted tumour tissue increase over several passages of PDTXs and a significant correlation was found between PDTX passage number and features of higher tumour grade (Pearson *et al*, 2016). This indicates that clonal selection occurs over passages of PDTXs.

The contribution of tumour stroma to tumour growth upon engraftment remains controversial in PDTX models. Components of human stroma, including vasculature, immune cells and fibroblasts, are present during early passages of PDTXs. The presence of these human stromal components allows for interaction studies between tumour cells and their microenvironment. However, the human stroma is subsequently replaced by murine stroma over several passages of PDTXs (Julien *et al*, 2012; Peng *et al*, 2013). Gene expression studies of NSCLC PDTXs confirmed depletion of human‐derived tumour‐associated cells with a downregulation of genes corresponding to cell adhesion and immune response pathways. This suggests that the PDTX deviates from the original tumour over time (Morgan *et al*, 2017). In addition, drug metabolism and pharmacokinetics differ between mouse and human, which needs to be taken into account (Morgan *et al*, 2017).

Over the years, it has become increasingly clear that orthotopic transplantation provide a more physiological PDTX than heterotopic (e.g. subcutaneous) engraftment. It was previously demonstrated that orthotopic transplantation can lead to local invasive growth and metastases, similar to those observed in patients (Dai *et al*, 2015; Hoffman, 2015). In orthotopic PDTXs, tumour‐host interactions can be investigated at the relevant location of primary and secondary tumour growth, as well as the development of metastases. In a comparison between orthotopic and subcutaneous xenografts of pancreatic ductal adenocarcinoma (PDAC), metabolic differences were found. These differences could be attributed to differences in tumour microenvironment caused by the different location of engraftment (Zhan *et al*, 2017). These results highlight the complexity of cancers as well as the importance of location and environment of the transplantation site. Nonetheless, while orthotopic PDTXs more accurately mimic the primary tumours by resemblance of the native microenvironment, this method is technically challenging and labour‐intensive. Therefore, most studies still use subcutaneous engraftment of tumour tissue.

### PDTO models

During the last decade, techniques to grow tissues *in vitro* in 3D as organotypic structures have been established. These so‐called organoids can be grown from adult and embryonic stem cells and are able to self‐organize into 3D structures that reflect the tissue of origin (for adult stem cell‐derived organoids), or to which the differentiation was directed (embryonic stem cell‐derived organoids) (for a review see Clevers, 2016). The first adult stem cell‐derived organoid cultures were established from Lgr5‐expressing mouse intestinal stem cells that were placed in conditions mimicking the intestinal stem cell niche (Sato *et al*, 2009). By providing R‐spondin‐1, epidermal growth factor (EGF) and Noggin, and embedment of the cells in an extracellular matrix‐providing basement membranes extract, the Lgr5‐expressing stem cells received the signalling cues necessary to self‐renew, proliferate and form differentiated offspring, resembling the intestinal epithelium (Sato *et al*, 2009).

Since then, organoid cultures have been established for a variety of human tissues, including lung (Hild & Jaffe, 2016; Tan *et al*, 2017; Sachs *et al*, 2019), colon (Sato *et al*, 2011), stomach (Bartfeld *et al*, 2015), liver (Huch *et al*, 2015), pancreas (Boj *et al*, 2015), prostate (Chua *et al*, 2014; Karthaus *et al*, 2014), kidney (Jun *et al*, 2018; Schutgens *et al*, 2019) and fallopian tube (Kessler *et al*, 2015). Moreover, organoid culture protocols have been established for patient‐derived tumour tissue as well. Human tumour organoids have been generated from colon (Sato *et al*, 2011; van de Wetering *et al*, 2015), pancreas (Boj *et al*, 2015; Huang *et al*, 2015), prostate (Gao *et al*, 2014; Drost *et al*, 2016), breast (Sachs *et al*, 2018), gastric (Nanki *et al*, 2018; Yan *et al*, 2018), lung (Sachs *et al*, 2019), oesophageal (Li *et al*, 2018), bladder (Lee *et al*, 2018; Mullenders *et al*, 2019), ovarian (Kopper *et al*, 2019), kidney (Schutgens *et al*, 2019) and liver (Broutier *et al*, 2017; Li *et al*, 2019) tumour tissue (Fig 3). An important feature of a number of these PDTOs is that they genetically and phenotypically mirror the tumour epithelium, including its intra‐tumour heterogeneity (Huang *et al*, 2015; van de Wetering *et al*, 2015; Nanki *et al*, 2018; Sachs *et al*, 2018; Yan *et al*, 2018). In a recent study, Roerink and colleagues characterized organoids derived from single cells from several colorectal cancers (CRC) and showed extensive mutational diversification as well as differences in responses to anti‐cancer drugs between even closely related cells of the same tumour (Roerink *et al*, 2018). PDTO models show improved resemblance to the original tumour compared to 2D cultured cancer cell lines. Thereby, organoid cultures bridge the gap between *in vitro* 2D cancer cell line cultures and *in vivo* PDTXs (Sachs & Clevers, 2014; Drost & Clevers, 2018). Importantly, they can be expanded long term and cryopreserved, allowing for the generation of living tumour organoid biobanks (Weeber *et al*, 2015; van de Wetering *et al*, 2015; Fujii *et al*, 2016; Schütte *et al*, 2017; Li *et al*, 2018; Nanki *et al*, 2018; Sachs *et al*, 2018, 2019; Seino *et al*, 2018; Tiriac *et al*, 2018; Yan *et al*, 2018). So far, the majority of established PDTO cultures originate from epithelial cancers (carcinomas). Although most common adult cancers are carcinomas and epithelial in origin, a number of cancers are not, such as sarcomas, leukaemia and lymphomas. This remains a major challenge in organoid technology and is in contrast to PDTX models, which allow for the growth of a broad variety of cancers. While organoid cultures cannot mimic vasculature and tumour–stroma interactions, patient‐derived tumour organoids are a promising tool for several translational applications, such as high‐throughput drug screens and personalized medicine in a patient‐derived manner.

## Translational applications of PDTX and PDTO model systems

Most preclinical anti‐cancer agents entering clinical trials fail to acquire regulatory approval due to insufficient safety or inefficacy. This highlights the limitations of the predictive value of current preclinical models. However, in a study where they evaluated the therapeutic relevance of PDTXs, a panel of six human small‐cell‐lung carcinoma (SCLC) xenografts was treated with topotecan, a topoisomerase I inhibitor, combinations of topotecan and the topoisomerase II inhibitor etoposide or alkylating agents ifosfamide or cisplatin at maximum tolerated dose (Némati *et al*, 2010). Three out of the six PDTX models showed over 90% growth inhibition when treated with topotecan alone, similar to the therapeutic response observed in Phase II clinical trials (Ardizzoni *et al*, 1997). Growth inhibition in the PDTX models was improved when topotecan was combined with etoposide or ifosfamide. These findings demonstrate that the established xenografts are useful for preclinical assessment of new drugs and combinations of drugs (Némati *et al*, 2010; Rosfjord *et al*, 2014). Moreover, Bertotti and colleagues screened a cohort of 85 metastatic CRC (mCRC) PDTX models, treated with cetuximab, an inhibiting antibody against epidermal growth factor receptor (EGFR). They found an enrichment of tumours with HER2 amplification in cetuximab‐resistant *KRAS*/*NRAS*/*BRAF*/*PIK3CA* wild‐type tumours (Bertotti *et al*, 2011). This proof‐of‐concept study revealed that the combined inhibition of EGFR and HER2 induced long‐lasting tumour regression, suggesting promising therapeutic opportunities for mCRC patients that are resistant to cetuximab (Bertotti *et al*, 2011).

Gao and colleagues generated an extensive collection of more than 1,000 PDTX models representing a broad range of solid cancers. In this large panel of PDTX models, genetic hypotheses and biomarkers of sensitivity to cancer treatments, derived from cultured cancer cell lines, were successfully validated. Importantly, the PDTX models also identified therapeutic candidates that cancer cell lines failed to capture (Gao *et al*, 2015; Byrne *et al*, 2017). The promising results of such large cohort studies increase the use of PDTXs for preclinical models of testing anti‐cancer drugs and to unravel biomarkers for drug sensitivity and resistance. For example, a recent study demonstrated that metformin, an anti‐diabetic drug, also affects tumour growth in CRC PDTX models. Administering metformin at physiological levels of 150 mg/kg per day in mice, which is equivalent to the clinical dose of 500–1,000 mg/daily in human, is sufficient to inhibit tumour growth in CRC PDTX. This implies promising therapeutic options for CRC patients (Suhaimi *et al*, 2017). Together, these studies demonstrate the promises of PDTXs as preclinical models to develop novel cancer treatments and predict their clinical response in patients.

Fast expansion of preclinical model systems is important to enable high‐throughput drug screens. In contrast to PDTXs, patient‐derived organoid cultures can be more easily expanded long term and several studies demonstrated that organoid cultures allow for the detection of gene–drug associations and enable high‐throughput drug screens. Verissimo and colleagues tested KRAS pathway inhibitors and combinations of drugs on normal colon organoids and CRC PDTOs and demonstrated that only organoids harbouring KRAS mutations were resistant to the treatments (Verissimo *et al*, 2016). In another recent study, Vlachogiannis and colleagues reported a living biobank of PDTOs from metastatic, heavily pretreated colorectal and gastroesophageal tumours, which showed a high degree of similarity to the original patient tumours. A comparison of responses to anti‐cancer agents in PDTOs and PDTO‐based orthotopic mouse tumour xenograft models with the responses of the patients in clinical trials, suggests that PDTOs successfully recapitulate the response seen in the patient (Vlachogiannis *et al*, 2018). Additionally, Tiriac and colleagues generated a pancreatic cancer PDTO library that largely maintained the mutational spectrum and transcriptional subtypes of primary pancreatic cancer. They showed that pancreatic cancer PDTOs exhibited heterogeneous responses to standard‐of‐care chemotherapeutics and that these therapeutic profiles correspond to patient outcomes. These data suggest that combined molecular and therapeutic profiling of PDTOs may predict treatment response and enable prospective therapeutic selection (Tiriac *et al*, 2018). Moreover, Schütte and colleagues collected a large biobank of 106 CRCs, 35 PDTOs and 59 PDTXs to identify novel biomarkers by linking molecular profiles with drug sensitivity patterns. Although the genetic landscape of the original tumours was largely maintained, they also found some differences between PDTXs and PDTOs, as PDTXs appeared closer to the molecular distinct CRC groups than PDTOs. Additionally, PDTOs showed elevated expression levels of genes involved in xenobiotic and fatty acid processes, which may affect drug sensitivity (Schütte *et al*, 2017).

Organoid cultures additionally allow for genetic engineering to study effects of oncogenic mutations in detail. Li and colleagues introduced oncogenic mutations into primary organoids from mouse colon, stomach and pancreas. This study shows that pancreatic and gastric organoids presented dysplasia as a result of the activating *Kras* ^G12D^ mutation, loss of *Tp53* or both and formed adenocarcinoma upon *in vivo* transplantation. In contrast, primary colon organoids required combinatorial *Apc*,* Tp53, Kras* ^G12D^ and *Smad4* mutations for the formation of adenocarcinoma *in vivo*. Opposed to colon organoids, small intestine organoids showed more rapid dysplasia even with only the combination of mutated *Apc* and *Kras* or mutated *Apc* and *Tp53* (Li *et al*, 2014). Subsequently, two studies translated this to the human situation by CRISPR/Cas9‐mediated genome editing of common CRC driver mutations in healthy human small intestinal and colonic organoids (Drost *et al*, 2015; Matano *et al*, 2015). These studies demonstrated that organoids harbouring an activating mutation in *KRAS*, in combination with inactivating mutations in *APC*,* TP53* and *SMAD4*, are able to grow independent of the intestinal stem cell niche factors EGF, Wnt, R‐spondin and Noggin. Additionally, Drost *et al* showed that loss of *APC* and *TP53* are key drivers of chromosome instability and aneuploidy (Drost *et al*, 2015). Not only adult stem cell‐derived organoids can be used to study cancer initiation and progression, but also embryonic stem cell‐derived organoids can be valuable tools in cancer research. Huang *et al* directed the differentiation of human embryonic stem cells towards pancreatic progenitor cells that formed ductal and acinar structures. Expression of mutant KRAS or TP53 in progenitor organoids induced mutation‐specific phenotypes. For instance, mutated *TP53*, but not mutated *KRAS*, induced cytosolic SOX9 localization, which was associated with mortality of patients (Huang *et al*, 2015). In another recent study, Drost *et al* used CRISPR‐modified human stem cell organoids to study DNA repair defects in cancer (Drost *et al*, 2017). This showed that accumulation of mutations in organoids deficient in the mismatch repair gene *MLH1* accurately models the mutation profiles observed in mismatch repair‐deficient CRC. Application of this approach to the cancer predisposition gene *NTHL1* demonstrated that a high contribution of a mutational footprint (signature 30), observed in a breast cancer cohort, within a tumour can be indicative of germline mutations in *NTHL1* (Drost *et al*, 2017). These studies demonstrate the immense opportunities organoid technology gives to study the effects of specific genetic alterations during cancer initiation and progression.

## Integrating the tumour environment in PDTX and PDTO

The location in which a tumour resides, the tumour niche or microenvironment, plays an important role in cancer development. Stromal cells not only modulate the behaviour of tumour cells directly but are also able to influence the immune system (Tauriello *et al*, 2018). This is effectively shown in a study by Batlle and colleagues, using a mouse model in which the main four CRC driver mutations can be specifically modified in intestinal stem cells. Quadruple‐mutant mice developed metastatic tumours in the small and large intestine that showed hallmarks of human CRC, including T‐cell exclusion and TGFβ‐activated stroma. They showed that inhibition of TGFβ induced a cytotoxic T‐cell response against tumour cells that prevented metastasis, highlighting the importance of the tumour microenvironment (Tauriello *et al*, 2018).

Immune cells recognize antigens present on cell membranes and distinguish between cancer cells and non‐cancer cells (Schumacher & Schreiber, 2015). As a consequence of tumour‐specific mutations, cancer cells start expressing neoantigens on their membranes, which can be recognized by T lymphocytes. The recognition of such neoantigens is an important factor in the efficacy of clinical immunotherapies. Additionally, the neoantigen load may form a biomarker for cancer cells (Schumacher & Schreiber, 2015). However, the lack of an immune competent environment in both immune‐deficient mice and organoid cultures limits the utility of these models to explore the interaction between a tumour cell and the immune system. To overcome this limitation in PDTX models, humanized mouse models have been developed (Box 1). For this, selected immune components were introduced to establish a competent human immune system (HIS) in mice. Humanized mice maintain various lineages of human blood cells throughout the lifetime of the recipient animal. Ideally, the hematopoietic stem cells (HSCs) come from the same patient from whom the PDTX will be established, in order to avoid immune reactions caused by human leucocyte antigen mismatch. This is challenging, because bone marrow biopsies are a burden for weakened patients. Additionally, growth factor‐stimulated bone marrow mobilization for collecting HSCs from peripheral blood might support tumour progression (Voloshin *et al*, 2011). The low yields of CD34‐positive HSCs obtainable from cancer patients strongly limit the number of humanized mice. Nonetheless, humanized mice allow studying features of the human anti‐tumour immune response in a mouse model system (Shultz *et al*, 2012; Byrne *et al*, 2017). In such a study, newborn NSG mice were co‐engrafted with human HSCs and human breast cancer cells. In these mice, tumour growth was accompanied by specific T‐cell maturation as well as tumour cell‐specific activation of T cells. Additionally, an accumulation of NK cells was observed at the tumour site (Wege *et al*, 2011). Transplantation of primary lung tumours into humanized mice revealed the existence of tumour‐infiltrating effector memory T cells that were activated upon human IL‐12 administration (Simpson‐Abelson *et al*, 2008). The ability to study tumour progression combined with its engrafted immune system provides new approaches for cancer immunotherapy, resistance of tumour cells to anti‐cancer therapies and the involvement of the immune system in response to chemotherapy.

In tumour organoid cultures, the lack of an immune competent environment can be overcome by co‐culture with immune cells. Nozaki and colleagues developed a novel co‐culture system of mouse intra‐epithelial lymphocytes and intestinal epithelial cells. In this co‐culture, the intra‐epithelial lymphocytes were expanded with intestinal organoids in the presence of IL‐2, IL‐7 and IL‐15 (Nozaki *et al*, 2016). Recently, Zumwalde *et al* (2016) succeeded in characterizing the intra‐epithelial lymphocyte compartment of healthy human breast tissue as well as identifying a subset of T lymphocytes that can be pharmacologically targeted to enhance their response to breast cancer cells. Specifically, Vδ2^+^ γδ T cells were constantly present in the preparation of mammary ductal epithelial organoids. In response to zoledronic acid, an aminobisphosphonate drug, these T lymphocytes started to proliferate. Additionally, Vδ2^+^ T cells from breast ductal organoids produced IFNγ, an anti‐tumour cytokine, and efficiently killed bisphosphonate‐pulsed breast cancer cells. Together, these results demonstrate the potential for Vδ2^+^ γδ T lymphocytes to respond to FDA‐approved bisphosphonate drugs as a novel immunotherapeutic approach to inhibit cancer growth (Zumwalde *et al*, 2016). In another recent study, Dijkstra and colleagues established and validated a platform to induce and analyse tumour‐specific T‐cell responses to epithelial cancers, including mismatch repair‐deficient CRC and NSCLC, in a personalized manner. Enrichment of tumour‐reactive T cells from peripheral blood of patients was successfully established by co‐cultures of peripheral blood lymphocytes with autologous tumour organoids. Moreover, they demonstrated that these tumour‐reactive T cells efficiently recognize and kill autologous tumour organoids, while leaving the healthy organoids or tissue unharmed (Dijkstra *et al*, 2018). In addition, Neal *et al* (2018) developed an air–liquid interface PDTO culture system that recapitulates complex tumour architecture including stromal and immune compartments. They demonstrated that the T‐cell receptors are highly conserved between the PDTO culture and the parental tumour. Crucially, they showed that the PDTO cultures functionally recapitulate the PD‐1/PD‐L1‐dependent immune checkpoint (Neal *et al*, 2018).

In addition to the immune system, cancer‐associated fibroblasts (CAFs) play an important role in the tumour environment. As such, Öhlund and colleagues showed that a co‐culture of murine pancreatic stellate cells (PSCs) and PDAC tumour organoids recapitulate properties of PDAC desmoplasia. They demonstrate that PSCs differentiate into two distinct subtypes of CAFs with elevated expression of αSMA and secretion of IL‐6 and additional inflammatory mediators, respectively (Öhlund *et al*, 2017). In accordance with this, Seino and colleagues established a co‐culture of PDAC organoids and CAFs and showed that the CAFs provide a WNT niche for PDAC (Seino *et al*, 2018), highlighting the importance of CAFs in the tumour microenvironment. In an effort to model diabetic vasculopathy *in vitro*, a recent study reported the development of human blood vessel organoids from pluripotent stem cells that self‐assemble into capillary networks, containing endothelial cells and pericytes, surrounded by a basement membrane. Upon transplantation of these organoids into mice, a perfused vascular tree is formed, including arteries, arterioles and venules (Wimmer *et al*, 2019). These human blood vessel organoids may open new doors for PDTO co‐cultures. Culturing PDTOs in the presence of the vascular system, in addition to co‐cultures with CAFs and immune cells, can recapitulate more components of the tumour microenvironment *in vitro*.

In conclusion, the lack of an immune competent environment in both PDTX and PDTO model systems can be overcome by using humanized mouse models or generating co‐cultures with immune cells, respectively. Additionally, to mimic the tumour stroma in the organoid model system, PDTOs can be co‐cultured with CAFs.

**Box 1: Humanized mouse models**

Humanized mice are immunodeficient mice engrafted with human hematopoietic stem cells which give rise to a variety of human blood cell lineages throughout the life of the animal. The human immune system (HIS) mouse model can be generated by transplantation of CD34‐positive human hematopoietic stem cells (HSCs) or precursor cells. These cells can be isolated from bone marrow, peripheral blood or umbilical cord blood. CD34‐positive HSC transplantation can be performed alone or in combination with transplantation of human immune tissues, such as thymic tissue (Drake *et al*, 2012). Humanized mouse models are powerful tools for studying cancer, haematopoiesis, and inflammatory and infectious disease.

## Drug screens and personalized medicine using PDTX and PDTO models

The discovery of molecular biomarkers for drug sensitivity is of high importance for the treatment of cancer patients. As illustrated by the EGFR tyrosine kinase inhibitor gefitinib in NSCLC, some drugs can give exceptional responses in small subsets of patients. However, when these patients are not properly identified within larger cohorts, it results in an overall negative clinical trial outcome (Thatcher *et al*, 2005). PDTX models can be used as screening platforms for predicting clinical outcome of the response rate to drugs, identifying biomarkers for drug sensitivity and studying drug resistance. For example, a prospective study in PDAC showed that the combination of the anti‐microtubule agent nab‐paclitaxel and the anti‐metabolite gemcitabine is effective in PDTX models of PDAC, which correlated with the clinical efficacy of this combination. Moreover, this combination of chemotherapeutics has been demonstrated to provide a survival benefit for advanced PDAC patients in a randomized phase III study (Von Hoff *et al*, 2013; Hidalgo *et al*, 2015). Additionally, PDTX models are not only able to provide potential clinical indications, but they may also facilitate the identification of potential drug efficacy biomarkers. In CRC for example, several studies have shown that *KRAS* mutant PDTX models do not respond to cetuximab. The wild‐type status of *KRAS* is now a well‐documented clinical biomarker for this targeted therapy (Hidalgo *et al*, 2015). Furthermore, melanoma PDTX models were involved in the identification of a mechanism of resistance to targeted drugs, such as the BRAF^V600E^ inhibitor vemurafenib. Additionally, a novel drug administration strategy that is clinically applicable was proposed to overcome resistance (Das Thakur *et al*, 2013). Although PDTX models are useful for low‐throughput drug screens and predicting clinical outcome, they do not allow for high‐throughput drug screens.

In contrast to PDTX models, PDTOs can be established and expanded more efficiently, which allows them to be used in medium‐ to high‐throughput drug screens. The use of PDTOs as a preclinical model for finding biomarkers and performing genotype–drug associations is just starting to be explored. A few studies already show promising results. For example, drug testing on a panel of organoids derived from 20 chemo‐naive CRC patients confirmed known drug sensitivity–genotype correlations. This proof of principle highlighted the potential of screening organoid biobanks to detect novel genotype–phenotype correlations (van de Wetering *et al*, 2015). In a recent study, Pauli and colleagues collected 145 specimens, representing 18 different tumour types derived from patients with metastatic solid tumours. PDTO cultures were established from 56 of these specimens (38.6%), which were obtained from biopsies or surgical resection and stored in a living biobank (Pauli *et al*, 2017). PDTOs of four patients were used to perform drug screens, targeting mutated pathways that were identified using whole exome sequencing (WES). PDTX models were subsequently used for the validation of the compounds that affected PDTO growth. They found that two patients, suffering from uterine carcinosarcoma and endometrial adenocarcinoma, respectively, carried similar driver mutations in *PIK3CA* and *PTEN*. Yet, the drug response profiles clearly distinguished the two patients. For the uterine carcinosarcoma case, they identified the combination of the PIK3 inhibitor buparlisib (Armstrong *et al*, 2017) with the hypoxia signalling suppressor vorinostat (Zhang *et al*, 2017) as one of the top drug combinations. By contrast, for the endometrial adenocarcinoma case, a combination of buparlisib with the PARP and HDAC inhibitor olaparib (Yuan *et al*, 2017) was found as optimal treatment in both PDTO and PDTX models. The latter is of high relevance, as no targeted therapies are approved for endometrial cancer yet (Pauli *et al*, 2017). The combination of WES, PDTO and PDTX models makes it possible to compare the efficacy of specific drugs on individual tumours thereby providing recommendations for patient care in a personalized manner. Moreover, it enables assessing how individual tumours evolve in response to therapies as well as determining next therapeutic steps for cases where standard clinical options have already been exhausted. Additionally, the combination of these techniques allows creating a database that relates drug sensitivity to tumour genetics. This enables to nominate potential therapeutic strategies even when only genomic data are available (Pauli *et al*, 2017). In conclusion, combining different techniques and model systems for cancer research can improve the prediction of clinical response to anti‐cancer treatments in a personalized manner.

## Outlook and challenges

The main challenge in preclinical cancer research remains the establishment of models that recapitulate the patient situation as close as possible, retaining intra‐tumour heterogeneity and the tumour environment. So far, not all cancer types can be grown in mice or as tumour organoids. For the establishment of more PDTO models, it is of high relevance to find the optimal *in vitro* growth conditions that enable tumour cells to grow, while keeping as much cellular heterogeneity as possible. Selection pressure occurs in both PDTX and PDTO model systems (Morgan *et al*, 2017; Pauli *et al*, 2017). Therefore, combining the strengths of both preclinical model systems will be powerful for investigating therapeutic responses. For example, PDTOs can be used for high‐throughput drug screenings and selecting effective drugs or drug combinations. Subsequently, the efficacy of these selected drugs should be validated in PDTX models (Pauli *et al*, 2017). Together, these preclinical models can reflect the response to anti‐cancer therapies and give indications for patient‐tailored treatment.

## Conclusions

In this review, we discussed the role of PDTX and PDTO model systems in cancer research and therapy development (Table 1). While PDTXs have already been established for a broad variety of cancers (Fig 3), the fast‐evolving improvements in PDTO culture systems hold great promise. Transplanting primary tumour tissue directly into mice allows for the partial resemblance of the tumour mirco‐environment, including stromal components, such as CAFs and vasculature. Although PDTO cultures do—so far—not maintain the stromal components of human tumours, they represent genetic and phenotypic heterogeneity found in human cancers. Additionally, organoid cultures can be expanded relatively fast, cryopreserved and genetically modified. These features allow for generating living tumour organoid biobanks and providing a platform for high‐throughput drug screens. Although both models lack an immune competent environment, this limitation can be overcome by transplantation of HSCs and co‐culture with T lymphocytes for PDTX and PDTO models, respectively.

**Table 1:** Characteristics of patient‐derived tumour xenograft (PDTX) and tumour organoid (PDTO) model systems. Features are rated as best (++), suitable (+), possible (−/+) and unsuitable (−) (adapted from Sachs & Clevers, 2014)

Feature	PDTX	PDTO
Ease of use	+	++
Initiation efficiency	+	++
Scalability	+	++
Genetic stability	++	++
Intra‐tumour heterogeneity	++	+
Genetic modification	−/+	++
Integratable immune system	−/+	−/+
Tumour–stroma interaction	+	−/+
Low‐throughput drug screens	+	++
High‐throughput drug screens	−	++
Prediction of clinical response	++	++
Testable drug classes^a^	3	2

a Drug classes: (1) agents targeting tumour‐specific proteins, (2) agents targeting host–tumour interactions and (3) agents targeting tumour cells empirically.

In addition to PDTX and PDTO, several other preclinical models have been developed over the years. Induced pluripotent stem cells (iPSCs) can be differentiated towards several lineages and can be used to model normal development *in vitro*. iPSCs were successfully used for exocrine differentiation of pancreatic progenitors and for modelling PDAC tumour organoids (Huang *et al*, 2015) as well as for differentiation to colonic organoids for the modelling of CRC (Crespo *et al*, 2018). However, two main challenges in the establishment of iPSC‐derived cancer models are the efficiency of malignant cell reprogramming and the capability to differentiate the iPSCs into the cell lineage of interest (Papapetrou, 2016). Additionally, during the establishment of iPSC‐derived cancer models, selective outgrowth of tumour sub‐clones may occur when a subset of cells harbours specific mutations resulting in a loss of heterogeneity. This is in stark contrast to PDTO model systems that largely recapitulate the genetic heterogeneity of the parental tumour (Huang *et al*, 2015; van de Wetering *et al*, 2015; Nanki *et al*, 2018; Sachs *et al*, 2018; Yan *et al*, 2018). Another recent study described a technology termed conditional reprogramming, which allows efficient establishment of patient tissue‐derived 2D cancer cell cultures in the presence of RHO kinase inhibitor and a fibroblast feeder layer (Liu *et al*, 2012). It is of high importance to combine multiple cancer models to get the best possible prediction of tumour sensitivity to and toxicity of anti‐cancer treatments, which will ultimately result in more efficient translation from bench to bedside.

## Conflict of interest

The authors declare that they have no conflict of interest.
